# Respiratory health effects of the fiberglass-reinforced plastic lamination process in the yacht-building industry

**DOI:** 10.5271/sjweh.3924

**Published:** 2020-12-16

**Authors:** Chi-Hsien Chen, Perng-Jy Tsai, Ya-Fen Wang, Chih-Hong Pan, Po-Chen Hung, Jiune-Jye Ho, Diahn-Warng Perng, Benoit Nemery, Yue Leon Guo

**Affiliations:** 1 Department of Environmental and Occupational Medicine, National Taiwan University (NTU) Hospital Hsin-Chu Branch, Hsinchu, Taiwan; 2 Department of Environmental and Occupational Medicine, NTU College of Medicine and NTU Hospital, Taipei, Taiwan; 3 Department of Environmental and Occupational Health, National Cheng Kung University, Tainan City, Taiwan; 4 Environmental Engineering, Chung Yuan Christian University, Taoyuan City, Taiwan; 5 Institute of Labor, Occupational Safety and Health, Ministry of Labor, New Taipei City, Taiwan; 6 School of Medicine, National Yang-Ming University, Taipei City, Taiwan; 7 Department of Chest Medicine, Taipei Veterans General Hospital, Taipei City, Taiwan; 8 Department of Public Health and Primary Care, Centre for Environment and Health, KU Leuven, Leuven, Belgium; 9 Institute of Environmental and Occupational Health Sciences, NTU College of Public Health, Taipei, Taiwan

**Keywords:** induced sputum, laminating process, laminator, lung function, respiratory symptom, styrene

## Abstract

**Objectives:**

Fiberglass-reinforced plastics (FRP) manufacturing has been related to cases of severe airway obstruction and elevated risk of respiratory mortality. But the specific job content risk is not clear. This study evaluated the respiratory health effects of the FRP lamination process.

**Methods:**

A questionnaire was used to evaluate respiratory symptoms of workers in two yacht-building plants. Pre-shift (07:30–08:30 hours) and post-shift (17:00–18:00 hours) lung function was measured, while post-shift induced sputum was collected on the first day of the week. The participants were grouped into FRP laminators and non-laminators. Linear and logistic regression was used to investigate the effects of the lamination process on lung function.

**Results:**

Laminators had a higher prevalence of chronic cough, lower pre-shift forced expiratory volume in first second (FEV1) and FEV1/force vital capacity (FVC) (-3.3% and -1.5%), lower post-shift FVC and FEV1 (-3.6% and -4.9%), and larger post-shift reduction of FVC (-2.1%) compared to non-laminators. The laminators also had higher risk of early obstructive and overall (obstructive plus restrictive) lung function impairment, and post-shift reduction of FVC >10% [odds ratio (OR) 5.98, 4.98, and 3.87, respectively). They also had higher percentages of neutrophils and lymphocytes in the induced sputum.

**Conclusion:**

Laminators should undergo regular check-ups of respiratory symptoms and lung function. Further toxicologic studies are warranted to identify the specific causal agent in the FRP lamination process.

In 2013, eight workers involved in laying up fiberglass woven roving with polyester resins were reported to have obliterative bronchiolitis, a severe irreversible airway obstructive disease involving the small bronchioles (1, 2). Two of them received lung transplantation and one died while waiting for an organ donor. These cases echoed findings of past cohort studies. Two studies that followed up a large cohort of workers in 30 reinforced plastics manufacturing plants in the United States found elevated mortality risk due to non-malignant respiratory diseases, particularly obstructive airway diseases (3, 4). Another cohort study reported elevated mortality risk due to the code “Pneumoconiosis and other respiratory diseases” from the US reinforced plastic boat-building industry (5). However, a Danish cohort did not show the same risk (6).

Aside from case reports and death registry analysis, epidemiologic surveys using lung function tests and other biomarkers can provide pathophysiologic information for non-diagnosed and subclinical cases. Epidemiologic studies on the respiratory health of workers in fiberglass-reinforced plastics (FRP) manufacturing remain limited. Because styrene is one of the most abundant volatile compounds used in FPR and is the active diluent of polyester resin (36–45% of weight concentration), it is a major concern (7). Previous evidence on the effects of styrene exposure on lung function are mixed. Helal et al (8) and McCague et al (9) both report an association of styrene exposure and obstructive lung function impairment among workers in FRP manufacturing plants. However, Lorimer et al (10) do not report either obstructive or restrictive lung function impairment among workers involved in styrene monomer or polymer fabrication. Whether the observed lung function effects are related to styrene or the additives used in FRP is unknown. Another study reports on increased lung inflammation among workers exposed to FRP dusts related to cutting, grinding, and finishing processes (11). What kind of work process is most hazardous to the lungs is still not clear.

Identifying the health risk of a specific group with similar exposure is an important step in occupational health. In the light of previous case reports, all of which involve manually wiping the glue and laying up the FRP, we conducted this study to evaluate the respiratory effects associated with the lamination process.

## Methods

### Design and study population

A cross-sectional study on two yacht-building plants in Taiwan was conducted in 2011 (plant A) and 2015 (plant B). All the employees (68 in the plant A and 54 in the plant B), including administrative personnel, were invited to participate. The Institutional Review Board of the National Taiwan University Hospital approved the study protocol (201110017RC), and each participant provided informed consent. Pre-shift (07:30–08:30 hours) and post-shift (17:00–18:00 hours) lung function was measured, and post-shift induced sputum was collected on Mondays – the first workday of the work-week. Each participant received two measurements of lung function and one collection of sputum. All of the participants answered a questionnaire that provided demographic information, respiratory symptoms, cigarette smoking habits, and medical diagnoses.

### Measurements of styrene concentrations

Aerial concentrations of styrene were measured during the study. In plant A, first, we performed stationary monitoring with the use of photo-ionized detector (PID) to measure concentrations of total volatile organic compounds near the lamination process paired with air sampling by canisters for 40 minutes. Canister-sampled air was further analyzed for concentrations of various chemicals, including styrene, by GC-MS. Eight repeats of PID-canister-paired monitoring were done, and the styrene estimation formula by PID values was obtained. Next, we measured total volatile organic compounds by PID for 10 minutes near the breathing zone of laminators who were doing lamination. A total of 36 person-times PID monitoring was done among 13 laminators. Their styrene exposures were then estimated by PID values. In plant B, styrene was sampled by personal active samplers with active carbon tubes for seven hours during work and analyzed by GC-FID method. We took 47 measurements, including 9 for lamination work, 12 for administration, 9 for woodwork, 6 for grinding work, and 11 for maintenance. Concentrations of styrene were expressed as parts per million (ppm) (1 ppm=4.26 mg/m^3^).

The processes of lamination, grinding, and woodwork were similar in the two plants, and employees involved in these three processes worked in the same building structure of each plant. Plant A and plant B were both semi-open. The employees involved in the lamination (laminators) usually stood inside the wooden yacht mold and repeatedly used rollers to impregnate the resin and rolled it onto the glass fiber roving layer-by-layer. Sometimes the laminators needed to enter more restricted compartments for lamination. Employees engaged in grinding work used a hand-held grinder to polish the surface of the hull. Woodworkers were responsible for the manufacture of wood-related furniture and facilities inside the hull. Woodworkers were usually exposed to wood dust and sometimes paint. The administrative staff’s office in plant A was located in a building separate from the yacht construction factory, while, in plant B, it was located in the same building structure. Maintenance workers were responsible for the maintenance of water, electricity, and various machinery and equipment in the factory, and their working place was not fixed inside the building of lamination work. Regarding personal protective equipment, plant A provided a half-face mask with a cartridge for the laminators, but plant B only used general cotton or activated carbon masks. During the surveys, due to the hot weather, many workers did not use the respirator correctly.

### Pulmonary function measurement

Spirometry was measured based on the guidelines of the American Thoracic Society (12). Each participant underwent lung function assessment in a sitting position. At least three forced expiratory maneuvers with smooth flow-volume loops without artifacts and <5% or 150 ml difference in lung volume between the best two blows were required. Forced vital capacity (FVC), forced expiratory volume in one second (FEV1), and the ratio of FEV1 to FVC (FEV1/FVC) were used for data analyses. Spirometer (Chest-graph HI-101; CHEST MI, Tokyo, Japan) was calibrated before each survey using a 3L flow-volume syringe. Lung function results were defined as obstructive if either pre- or post-shift FEV1/FVC was less than the lower limit of normal (LLN), and defined as restrictive if maximum FVC was less than LLN with normal FEV1/FVC. The predicted values of FVC and FEV1 and the LLN values of FEV1/FVC and FVC were calculated from the Global Lung Initiative 2012 equation, with ethnic adjustments for South East Asians (13).

### Sputum induction and processing

Sputum was induced through increasing concentrations of saline (eg, 3%, 4%, and 5%), as described by Perng et al (14). The procedure was performed after the lung function assessment. Only the opaque and dense portions of the induced sputum (mucus plugs) were selected and collected to minimize contamination by oropharyngeal secretion. The sputum was processed within four hours after collection. Due to the shortage of laboratory support, the preparation of a stained cytospin slide with the cell suspension of induced sputum and the assessment of its differential cell count were only performed in the survey of plant A. A sample was considered appropriate if squamous cells were <20% of the total cell count. If oral squamous cell contamination is <20%, the percentage of differential cell counts in induced sputum cell count is a widely used marker for phenotyping airway inflammation with good reproducibility (15). Since the selection and filtration process of sputum may cause deviations in the total cell number, resulting in unreliable absolute cell number (16), we select the percentages of differential cell counts for analysis in this study. The proportion of differential cell counts was calculated as a percentage of total inflammatory cell count.

### Statistical analysis

The participants were grouped into laminators and non-laminators (ie, administrative and maintenance staff, carpenters, and grinding workers). Between-group differences in demographic information and respiratory symptoms were analyzed by Chi-square, Fisher exact test, or Student’s t-test. Between-group differences in differential count in the induced sputum were analyzed by Wilcoxon rank sum test.

Linear and logistic regressions were used to evaluate the relationship between the FRP lamination process (laminators/non-laminators), tenure of lamination work (in years), lung function parameters, and patterns of ventilatory defects, with adjustments for age, sex, educational attainment, tenure, current smoking, past smoking, and cumulative smoking amount. To examine whether sputum differential cell counts could be an intermediate biomarker for functional phenotyping of the respiratory system, we used linear regression to assess the association between the percentage of each cell type with lung function parameters. Statistical significance was set at P<0.05.

## Results

Overall, the study participants consisted of 113 employees, 63 from plant A and 50 from plant B. The proportions of female workers and educational attainment <13 years were higher among the laminators than among the non-laminators (table 1). There were no significant differences in age, tenure, and smoking habits between the two groups. The proportion of current and past smokers was much higher among male (19.7%) than female (4.7%) workers.

**Table 1:** Demographic profile and prevalence of respiratory symptoms of the study participants (N=113). [SD=standard deviation.]

	Laminator (N=59)		Non-laminator (N=54)		P-value a
	
	Mean (SD)	N (%)	Mean (SD)	N (%)	
Age (years)	40.6 (10.9)		40.9 (12.2)		0.898
Job tenure (years)	7.6 (7.2)		6.4 (8.0)		0.426
Smoking (pack-years) b	14.4 (8.2)		18.0 (15.0)		0.381
Female		32 (54.2)		11 (20.4)	0.0002
Current smoker		11 (18.6)		14 (25.9)	0.352
Ex-smoker		6 (10.2)		7 (13.0)	0.642
Education <13 years		28 (47.5)		12 (22.2)	0.005
Usual cough without airway infection		11 (19)		4 (7)	0.079
Daily timing of cough					
Early morning after wakeup		3 (5)		1 (2)	0.353
During work at daytime		6 (10)		0 (0)	0.028
After work in the evening		1 (2)		2 (4)	0.605
Before sleep at night		8 (14)		1 (2)	0.033
Within 2 hours after sleep		2 (3)		1 (2)	1
Before awaken		1 (2)		1 (2)	1
Chronic cough >3 months in past year		6 (10)		0	0.028
Usual phlegm		7 (12)		6 (11)	0.901
Daily timing of phlegm					
Early morning after wakeup		5 (8)		5 (9)	0.883
During work at daytime		1 (2)		2 (4)	0.605
After work in the evening		0		2 (4)	0.226
Before sleep at night		1 (2)		0	1
Within 2 hours after sleep		0		0	na
Before awaken		0		0	na
Chronic phlegm >3 months in past year		5 (8)		3 (6)	0.719
Ever wheeze with short of breath after doing this job		4 (7)		1 (2)	0.366
Daily timing of wheeze with short of breath					
Early morning after wakeup		0		0	na
During work at daytime		1 (2)		0	1
After work in the evening		0		0	na
Before sleep at night		3 (5)		0	0.245
Within 2 hours after sleep		0		1 (2)	0.478
Before awaken		0		0	na
Cough and wheeze improved during holiday		8 (14)		2 (4)	0.097
Usual nasal symptoms without airway infection		16 (27)		20 (37)	0.258

a P-values estimated by Chi square or Fisher exact test if expected value <5.

b Pack-years calculated only for workers with a history of smoking tobacco.

Geometric mean concentrations of styrene measured for laminating work amounted to 7.5 ppm in plant A and 16.5 ppm in plant B, ie, approximately 30–66 times higher than the geometric mean of 0.2–0.3 ppm measured (in plant B only) for the other jobs (table 2). In general, laminating in an enclosed space or over larger surfaces exposed workers to higher styrene concentrations, thus explaining the broad range of measured concentrations of 1.5–46 ppm in plant A and 2.7–71 ppm in plant B.

**Table 2:** Styrene exposure (ppm a) among workers doing different types of tasks b,c. [NM=not measured.]

	N	Mean	Geometric mean	Minimum	Maximum
Plant A					
Lamination	36	10.32	7.51	1.53	45.79
on-lamination			NM		
Plant B					
Lamination	9	29.47	16.46	2.69	70.70
Non-lamination	38	0.63	0.23	0.08	7.71
Administration	12	0.36	0.21	0.08	1.34
Wood work	9	0.35	0.18	0.09	1.82
Grinding work	6	1.37	0.21	0.10	7.71
Maintenance	11	0.74	0.32	0.09	3.51

a Conversion factor:1 ppm styrene = 4.26 mg/m^3^.

b Styrene concentrations were measured by static sampling in plant A for 40 minutes, and personal sampling in plant B for 7 hours.

c Occupational exposure limit (PEL-TWA) for styrene in Taiwan is 50 ppm.

Laminators had a statistically higher prevalence of chronic cough than non-laminators (table 1). Laminators more commonly complained of cough during work at daytime and before sleep at night. There was a borderline significance in the difference of cough and wheeze relieved during holidays between the two groups, but there was no significant difference in phlegm production and nasal symptoms.

The pre-, post-, and between-shift changes of lung function revealed that after adjustments for confounding factors, laminators had significantly lower pre-shift FEV1 and FEV1/FVC, lower post-shift FVC and FEV1, and larger post-shift reduction of FVC compared to non-laminators (table 3). In terms of patterns of lung function impairment (table 4), laminators had higher prevalences of obstructive and overall (obstructive plus restrictive) impairments. The prevalence of post-shift reduction of FVC >10% was also higher among laminators. The tenure for lamination work was negatively associated with pre- and post-shift FVC or FEV1 (supplementary material www.sjweh.fi/show_abstract.php?abstract_ id=3924, table S2). We further stratified all participants in to three groups, non-laminators (never) and those with lamination tenure < or >6.14 years (the median among laminators), and found that only those with lamination tenure <6.14 years had significantly higher risk of obstructive and overall (obstructive plus restrictive) impairments (table S3).

**Table 3:** Pre- and post-shift lung function in laminators and non-laminators. [CI=confidence interval; FVC=forced vital capacity; FEV1=forced expiratory volume in first second; SD=standard deviation.]

	Laminator	Non-laminator	Regression coefficient a					
		
	Mean (SD)	Mean (SD)	Crude	95% CI	P-value	Adjusted b	95% CI	P-value
Pre-shift lung function								
FVC (% of prediction)	93.0 (16.0)	95.4 (12.6)	-1.23	-3.92–1.47	0.369	-1.435	-4.35–1.48	0.332
FEV1 (% of prediction)	90.3 (15.5)	96.5 (14.9)	-3.12	-5.96–-0.29	0.031	-3.311	-6.42– -0.21	0.037
FEV1/FVC (%)	81.8 (6.8)	84.1 (6.7)	-1.18	-2.34–0.07	0.073	-1.529	-2.90– -0.16	0.029
Post-shift lung function								
FVC (% of prediction)	88.9 (14.5)	95.5 (14.7)	-3.32	-6.04–-0.60	0.017	-3.571	-6.57–-0.57	0.020
FEV1 (% of prediction)	87.7 (15.6)	96.5 (15.1)	-4.39	-7.26–-1.52	0.003	-4.879	-8.06– -1.70	0.003
FEV1/FVC (%)	82.9 (7.2)	84.2 (6.2)	-0.62	-1.88–0.65	0.337	-1.196	-2.55–0.16	0.083
Change of lung function (post - pre)								
FVC (% of prediction)	-4.1 (7.9)	0.1 (8.0)	-2.09	-3.57–-0.61	0.006	-2.136	-3.81– -0.46	0.013
FEV1 (% of prediction)	-2.6 (7.2)	-0.1 (9.7)	-1.26	-2.85–0.32	0.117	-1.567	-3.33–0.20	0.081
FEV1/FVC (%)	1.2 (6.9)	0.1 (6.4)	0.57	-0.68–1.81	0.371	0.333	-1.05–1.72	0.635

a The coefficient was estimated by linear regression modeling with non-laminator as the reference group.

b Adjusted for age, gender, education attainment, tenure, current smoking, past smoking, and cumulative smoking amount.

**Table 4:** Patterns of lung function impairment in laminators and non-laminators. [CI=confidence interval; FVC=forced vital capacity; FEV1=forced expiratory volume in first second; LLN=lower limit of normal.]

	Laminator	Non-laminator	Odds ratio a					
		
	N (%)	N (%)	Crude	95% CI	P-value	Adjusted b	95% CI	P-value
Obstructive c	14 (24)	4 (7)	3.89	1.29–14.49	0.024	4.11	1.17–17.59	0.037
Restrictive d	4 (7)	1 (2)	3.85	0.55–76.67	0.234	2.77	0.27–67.85	0.524
Obstructive or restrictive	18 (31)	5 (9)	4.30	1.56–13.94	0.008	3.61	1.13–13.34	0.038
Post-shift reduction >10%								
FVC	14 (24)	4 (7)	3.89	1.29–14.50	0.024	3.87	1.15–15.54	0.037
FEV1	7 (12)	2 (4)	3.50	0.80–24.23	0.129	4.88	0.97–37.84	0.077

a Odd ratio was estimated by logistic regression modeling with non-laminator as the reference group.

b Adjusted for age, gender, education attainment, tenure, current smoking, past smoking, and cumulative smoking amount.

c Definition of obstructive: FEV1/FVC <LLN.

d Definition of restrictive: FEV1/FVC >LLN and FVC <LLN.

Sputum was collected from 51 out of 63 participants in plant A: 12 could not produce sputum after hypertonic saline inhalation, while 2 were excluded due to inadequate sputum quality (squamous cell >20% of total cells). Laminators had higher percentages of neutrophils and lymphocytes, and lower percentage of macrophages than non-laminators (table 5). The percentage of neutrophils in the induced sputum was associated with the cross-shift reduction in FEV1 and FEV1/FVC, while the percentage of lymphocytes was negatively associated with post-shift FVC and FEV1 (table S1). The percentage of macrophages was positively associated with post-shift FEV1/FVC and cross-shift changes in FEV1 and FEV1/FVC (table S1).

**Table 5:** Percentages of differential cell counts in total inflammatory cells in induced sputum of laminators and non-laminators from plant A. [FRP=fiberglass-reinforced plastics; SD=standard deviation.]

%	FRP N=29	Non-FRP N=20	P-value a
	
	Mean (SD)	Mean (SD)	
Neutrophil b	47.8 (15.5)	38.6 (14.2)	0.024
Macrophage b	47.0 (15.4)	57.4 (13.8)	0.006
Lymphocyte b	5.0 (2.4)	3.6 (2.6)	0.048
Eosinophil b	0.3 (0.4)	0.4 (0.6)	0.983

a P-value was calculated by Wilcoxon rank sum test.

a The values of the percentage of differential cell counts were adjusted by current, past, and cumulative smoking amount.

## Discussion

In this cross-sectional study in two FRP manufacturing plants, we found that laminators chronically exposed to moderately high styrene levels exhibited an excess of respiratory complaints (mainly cough), impaired spirometry and signs of pulmonary inflammation when compared to non-laminators.

The prevalence of chronic cough (generally non-productive) was higher among laminators than non-laminators, even though the former had fewer smokers. Cough was a more common complaint during work at daytime and before sleep at night, but with recovery the next morning. Airway symptoms (cough or wheeze) also improved during holidays or days-off. These respiratory symptoms are suggestive of asthma and may explain why such affected workers were likely to be treated as asthmatic patients, thereby ending up with irreversible fixed airway obstructive disease, as noted in published reports (1, 2). Progressive dry cough, dyspnea, and occasional wheeze are the main symptoms of bronchiolitis obliterans (17, 18). In patients with flavoring-related bronchiolitis obliterans cough improved after leaving employment, but dyspnea did not disappear (18). Thus, regular check-up of respiratory symptoms, including persistent cough or cough with wheeze that are related to work, may provide useful information for identifying workers with significant pulmonary problems caused by exposure to the FRP lamination process.

The laminators had an elevated risk of obstructive lung function impairment. This is consistent with previous case reports and epidemiologic studies (1, 2, 8, 9). An epidemiologic study that used styrene as an indicator of exposure to FRP-related resins also demonstrated a higher risk of obstructive lung function impairment in the high exposure group (8, 9). However, styrene effect was not a consistent factor in other studies. A cohort study with analysis of mortality data revealed that increased risk of non-malignant respiratory death was not positively correlated to the cumulative dosage of styrene exposure (3). An epidemiologic study in a styrene producing industry also did not show a risk of obstructive or restrictive impairment due to styrene exposure (10). Although the present study does not tell us whether styrene or other chemicals is the causal agent, our findings point to laminating as the critical exposure. Consequently, laminators are the group requiring active preventive interventions.

Workers involved in the FRP lamination process using the open-molding method are usually exposed to the highest levels of evaporated chemicals from polyester resins (7). Aside from styrene, several chemicals are added to the resins. Peroxides like methylethylketone peroxide (1–1.5%) or benzoyl peroxide are used as initiators or catalysts to initiate the curing process. Peroxide catalysts require dimethyl aniline (0.1–0.3%) to be effectively activated at room temperature. Promoters such as cobalt naphthenate (0.3–1%) are also added to resins to increase the cure rate. However, no toxicologic information is available for these aforementioned additives in terms of respiratory toxicity or bronchiolitis obliterans. Regarding the filaments, the diameter of the fibers in the woven roving or mat used for the hand lay-up lamination of yacht construction is >10 micrometers, which makes it less likely to be directly inhaled into deep airways. Sometimes, the laminators need to eliminate air bubbles in the composite by hand-holding grinders, which break down glass fiber and resins into inhalable particles. But their overall dust exposure is not as high as that in grinding workers. Therefore, despite that we cannot completely exclude the possibility of filament-related respiratory effect, it is more likely other causal agents play more important roles. Although the causal agent remains unclear, applying closed molding, such as vacuum-assisted resin transfer molding, as well as adequately wearing respirators with organic vapor cartridges have been reported to be effective for reducing personal exposure to FRP-related chemicals (7).

Increased cross-shift reduction in FVC suggests restrictive lung function effects of acute exposure to FRP lamination-related substances. However, the pathophysiology is not identified or described in this study because of the lack of information on total lung capacity and post-bronchodilator lung function. It can only be speculated that the change may be related to processes in the small rather than large airways (mainly FEV1/FVC). Small airway diseases can present as a reduction in FVC due to occlusion or dynamic collapse of small bronchioles (19, 20). Some cases of bronchiolitis obliterans have restrictive or mixed restrictive and obstructive lung function impairment (18, 21). In the light of previous research on flavoring-related bronchiolitis obliterans, a survey using pre-bronchodilator spirometry has demonstrated a high prevalence of restrictive lung function impairment among flavoring workers (22). A spectrum ranging from obstructive to restrictive lung diseases is suggested for such toxic chemical inhalation (23). Therefore, FRP workers with either an obstructive or restrictive ventilatory defect on pre-bronchodilator spirometry need more detailed lung function assessments.

Susceptibility to bronchiolitis obliterans related to FRP chemicals is unknown, although some relevant clues have been provided in previous literature. First, only a small number of cases are reported to have severe obstructive lung disease and the symptoms often manifest shortly after employment (6–12 months), whereas other co-workers do not have the same symptoms or illness even under similar working conditions (1, 2). Second, there is a high risk of non-malignant respiratory mortality among FRP workers with less than one year of employment (5), and this risk is not related to cumulative exposure (3). Our research found that only workers with <6.14 years of employment in lamination have a higher risk of obstructive ventilatory disorder, which is consistent with previous research. Our study showed that a small percentage of workers experienced a cross-shift drop in lung function, suggesting an acute response. These “responders” may be the susceptible subgroup. Further longitudinal studies are needed to assess long-term respiratory outcomes among them.

The present study revealed an elevated percentage of sputum neutrophils rather than eosinophils among laminators. This is consistent with results by McCague et al (9) who showed suppressed exhaled nitric oxide levels, an indicator of eosinophilic airway inflammation. The association of neutrophils and cross-shift FEV1 and FEV1/FVC reduction also suggests a neutrophil-mediated response to airway injury and inflammation. In other words, sputum neutrophils might be used as an intermediate biomarker for the respiratory toxicity of FRP-lamination-related exposure. This finding is in line with previous literature showing that neutrophilic airway inflammation has been associated with toxic inhalation and chronic airway diseases, including bronchiolitis obliterans and chronic obstructive pulmonary diseases (24–26).

The elevated percentage of induced sputum lymphocytes among laminators, and its association with post-shift lung capacity, suggests that lymphocytes associated inflammation also play a role in the laminating related respiratory effect. A previous study has demonstrated an increased lymphocyte percentage in induced sputum in several interstitial lung diseases (27) in contrast to airway diseases like asthma or chronic bronchitis (28). Further animal studies or lymphocyte subset analysis may advance knowledge as to the pathophysiologic response to inhalational exposure in the lamination process.

The percentage of sputum macrophages in laminators was lower than that in non-laminators. Alveolar macrophages usually play a defensive role in respiratory exposure to pathogens and particulate matters (29). A previous study has shown an increase in the number of macrophages in bronchoalveolar lavage among workers exposed to FRP grinding dust (11). An animal study showed that wood dust could increase alveolar macrophages (30). Therefore, the observed reduction in its percentage in laminators may be related to lower dust exposure compared to employees involved in grinding and woodwork, and a relative increase in numbers of neutrophils and lymphocytes. The positive association between macrophage and cross-shift lung function change in this study also suggests a protective role of macrophages in respiratory physiology.

This study has some strengths. First, we conducted field research before and after work on Monday, which minimized the effect of exposures on the previous days. Therefore, the impact on preshift lung function is likely to be related to chronic exposures and the effect on cross-shift lung function changes linked to acute exposures. Second, the laminators who participated in this study usually remained in hand lay-up work in FRP manufacturing rather than moving around different kind of processes, which minimized the interference from other exposures, such as wood dust, painting, and FRP grinding dust. Third, this study included exposed and non-exposed groups in the same plant, which minimized bias related to environmental exposure (eg, ambient air pollution).

Some limitations are noteworthy. First, the study’s cross-sectional design may have caused an underestimation of the health hazards due to the healthy worker or healthy worker survival effect. Airway symptoms, such as cough or dyspnea, may alert workers to leave work. Since we have already observed the effects on lung function, the healthy worker effect may have prevented us from observing more severe cases of impaired respiratory function. Second, grouping laminators into a single exposure group may underestimate the health effects in the extremely high exposure groups, such as those more commonly working in an enclosed structure or with large surface lamination processes. The incomplete and differential measurement of styrene in the two plants prevented us from assessing the exposure–response relationship for the entire data set. Finally, as this is a simple cross-sectional study without long-term follow-up, reversed causality should be cautiously considered.

### Concluding remarks

Workers using the hand lay-up method for FRP lamination have higher risk of chronic cough, obstructive lung function impairment, cross-shift drop in lung function, and elevated neutrophilic and lymphocytic inflammation of the airways. Until the true causal agent used in the lamination process is identified and replaced, laminators should undergo regular health examinations with focus on respiratory symptoms and lung function. Workers should also use adequate personal protective equipment.

## Supplementary material

Supplementary tables
