# Supplementary material for: Respiratory health effects of the fiberglass-reinforced plastic lamination process in the yacht-building industry
Source: Scand J Work Environ Health. 2020 Dec 16;47(1):62–9. doi: 10.5271/sjweh.3924 (PMC7801144; doi:10.5271/sjweh.3924)
Supplement: Supplementary tables [file SJWEH-47-62-S001.pdf]

Respiratory health effects of the fiberglass-reinforced plastic lamination process in the yacht-building industry[1]

by Chi-Hsien Chen, MD, PhD, Perng-Jy Tsai, PhD, Ya-Fen Wang, PhD, Chih-Hong Pan, PhD, Po-Chen Hung, PhD, Jiune-Jye Ho, PhD, Diahn-Warng Perng, MD, PhD, Benoit Nemery, MD, PhD, Yue Leon Guo, MD, PhD [2]

1. *Supplementary tables*
2. *Correspondence to: Prof. Yue Leon Guo, Department of Environmental and Occupational Medicine, College of Medicine, National Taiwan University and National Taiwan University Hospital, Rm 339, 17 Syujhou Road, Taipei 100, Taiwan. [E-mail: leonguo@ntu.edu.tw]*

Supplement Table S1. Relationship between the percentages of differential cell counts in induced sputum and lung function in plant A (n=49) [a]

	Neutrophils%	Macrophages %	Lymphocytes%	Eosinophils%
Pre-shift adjusted lung function [b]				
FVC, % of predicted	-0.09 (0.15)	0.12 (0.15)	-1.27 (0.91)	5.01 (4.62)
FEV1, % of predicted	-0.09 (0.14)	0.13 (0.14)	-1.52 (0.85)	4.31 (4.41)
FEV1/FVC, %	0.02 (0.06)	-0.01 (0.06)	-0.20 (0.35)	-0.62 (1.75)
Post-shift adjusted lung function [b]				
FVC, % of predicted	-0.08 (0.15)	0.13 (0.15)	-2.10 (0.87) [c]	5.27 (4.61)
FEV1, % of predicted	-0.22 (0.14)	0.27 (0.14)	-2.07 (0.86) [c]	4.49 (4.54)
FEV1/FVC, %	-0.12 (0.05) [c]	0.12 (0.05) [c]	0.01 (0.30)	-0.21 (1.49)
Change of adjusted lung function (Post - Pre) [b]				
FVC, % of predicted	0.01 (0.08)	0.01 (0.08)	-0.83 (0.45)	0.26 (2.36)
FEV1, % of predicted	-0.13 (0.06) [c]	0.14 (0.05) [c]	-0.55 (0.35)	0.18 (1.81)
FEV1/FVC, %	-0.14 (0.05) [c]	0.13 (0.05) [c]	0.20 (0.33)	0.41 (1.69)

[a] The values in the table represent the correlation coefficients and standard errors, which were estimated by linear regression.

[b] Lung function parameters were adjusted for age, sex, educational attainment, tenure, current smoking, past smoking, and cumulative smoking amount.

[c] $p<0.05$

Supplement Table S2. The relationship between Pre- and Post-shift lung function and the tenure of lamination work. (n=113)

	Regression coefficient [a]					
	Crude	95% CI	P-value	Adjusted [b]	95% CI	P-value
Pre-shift lung function						
FVC, % of prediction	-0.44	(-0.86~-0.03)	0.036	-0.53	(-0.99~-0.07)	0.025
FEV1, % of prediction	-0.50	(-0.94~-0.06)	0.026	-0.60	(-1.10~-0.10)	0.020
FEV1/FVC, %	-0.07	(-0.27~0.13)	0.473	-0.07	(-0.29~0.16)	0.568
Post-shift lung function						
FVC, % of prediction	-0.68	(-1.10~-0.27)	0.002	-0.79	(-1.27~-0.32)	0.001
FEV1, % of prediction	-0.70	(-1.15~-0.26)	0.002	-0.81	(-1.32~-0.30)	0.002
FEV1/FVC, %	-0.05	(-0.24~0.15)	0.651	-0.03	(-0.25~0.19)	0.807
Change of lung function (Post - Pre)						
FVC, % of prediction	-0.24	(-0.47~-0.005)	0.050	-0.26	(-0.53~0.01)	0.061
FEV1, % of prediction	-0.20	(-0.45~0.05)	0.110	-0.22	(-0.50~0.07)	0.139
FEV1/FVC, %	0.03	(-0.17~0.22)	0.787	0.04	(-0.19~0.26)	0.739

[a] The coefficient was estimated for the effect per 1 year increase in lamination work by linear regression modeling.

[b] Adjusted for age, gender, education attainment, current smoking, past smoking, and cumulative smoking amount.

Supplement Table S3. The relationship between pattern of lung function impairment and the tenure of lamination work (n=113)

	Obstructive [a]			Restrictive [b]			Obstructive or restrictive		
	OR [c]	95% CI	P-value	OR [c]	95% CI	P-value	OR [c]	95% CI	P-value
Laminator tenure, yrs	1.34	(0.01~57.46)	0.89	2.19	(0.02~198.09)	0.723	3.89	(0.13~147.18)	0.432
Groups									
Non-laminators	1			-			1		
Laminator tenure <=6.14 yrs [d]	5.61	(1.50~24.99)	0.014	-	-	-	4.32	(1.20~17.42)	0.029
Laminator tenure >6.14 yrs [d]	1.86	(0.34~10.20)	0.463	-	-	-	2.76	(0.67~12.10)	0.162

[a] Definition of obstructive: FEV1/FVC <lower limit of normal (LLN)

[b] Definition of restrictive: FEV1/FVC >LLN and FVC <LLN

[c] Odd ratio was estimated by logistic regression modeling with adjustment for age, gender, education attainment, current smoking, past smoking, and cumulative smoking amount.

[d] Laminator tenure was stratified by median (6.14 years).
